# Editorial: Molecular Mechanisms and Physiological Significance of Organelle Interactions and Cooperation

**DOI:** 10.3389/fcell.2016.00145

**Published:** 2016-12-21

**Authors:** Michael Schrader, Markus Islinger

**Affiliations:** ^1^Department of Biosciences, College of Life and Environmental Sciences, University of ExeterExeter, UK; ^2^Institute of Neuroanatomy, Center for Biomedicine and Medical Technology Mannheim, University of HeidelbergMannheim, Germany

**Keywords:** membrane contact sites, organelle dynamics, peroxisomes, mitochondria, endoplasmic reticulum, intracellular signaling, organelle communication, lipid droplet

Compartmentalization is a characteristic feature of eukaryotic life. Subcellular compartments such as mitochondria, peroxisomes, or lipid droplets have long been regarded as isolated and static entities which are mainly defined by their protein composition and specific metabolic function. However, it is now evident that they undergo dynamic changes, share certain proteins and interact with each other, showing metabolic cooperation and cross-talk. Effective communication between organelles is essential for cell function, viability and response to external stimuli. Despite great advances in the identification and characterization of key components and molecular mechanisms associated with the biogenesis and function of organelles, information on how organelles interact and are incorporated into metabolic and signaling networks is just beginning to emerge. Organelle cooperation requires sophisticated targeting systems which regulate the proper distribution of shared proteins to more than one organelle. Organelle motility and membrane remodeling support organelle interaction and contact. This contact can be mediated by membrane proteins residing on different organelles which can serve as molecular tethers to physically link different organelles. They can also contribute to the exchange of metabolites and ions, or act in the assembly of signaling platforms. In this regard organelle communication events have been associated with important cellular functions such as apoptosis, antiviral defense, organelle division and biogenesis, ROS metabolism and signaling, and various metabolic pathways such as breakdown of fatty acids or ether lipid (plasmalogen) biosynthesis.

The goal of this Research Topic is to review, present, compare, and debate recent novel findings on the underlying molecular mechanisms and physiological significance of organelle interaction and cooperation with a particular focus on mitochondria, peroxisomes, endoplasmic reticulum, and lipid droplets and their impact on the regulation of cellular homeostasis. The special topic thus combines a set of reviews, perspectives and research articles.

Our understanding of how organelles physically interact and use cellular signaling systems to coordinate functional networks between each other is still in its infancy. Recent work on membrane contact sites, for example the mitochondria-ER associated membranes (MAM), deciphered molecular players enriched in membrane domains and is thereby beginning to reveal mechanistic insights into organellar interaction systems, allowing one organelle to influence the function of another. Identifying the key molecular players of such specialized membrane structures will be a prerequisite to understand how organelle communication is physically accomplished and will lead to the identification of new regulatory networks. The review by Schrader et al. provides a timely overview of organelle contact sites, the molecular components involved and discusses the potential functions of organelle interactions. I. Sparkes addresses the techniques applied to investigate membrane contacts with special emphasis on the use of optical tweezers for their biophysical characterization (Sparkes). Kunze and Berger review protein import mechanisms into different organelles, including mitochondria and peroxisomes, and their targeting signals. They focus on the similarity between N-terminal targeting signals, address dual targeting and bi-localization, and highlight its evolutionary relevance.

Wanders et al. focus on an important function of organelle interaction, namely the metabolic interplay between peroxisomes and mitochondria as well as the ER. They highlight the disease-relevant interplay between peroxisomes and mitochondria in the breakdown of fatty acids by β-oxidation, as well as the cooperative role of peroxisomes and the ER in the biosynthesis of ether lipids that are required for the myelin sheath in humans. An important question is how substrates and products of metabolic networks are exchanged between participating organelles. Gao and Goodman review our current knowledge on how and why cytoplasmic lipid droplets interact with other subcellular organelles. Important functions are seen in the transfer of lipids between compartments, the supply of lipids for membrane expansion, energy production, and signaling. The underlying mechanism and extent of activation of lipases by contact sites, and the mode of fatty acid transfer between organelles, still remain to be elucidated.

Mueller and Reski focus on mitochondrial dynamics and interactions in plants. Using the model moss *Physcomitrella patens*, they provide microscopic evidence for the existence of mitochondria-ER interactions in plants, their correlation with mitochondrial dynamics and a potential role for MELL1 in modulating mitochondrial association to the ER. In their research article, Woods et al. present experimental evidence for a role of the microtubule cytoskeleton and the protein CluA in mitochondrial dynamics in the soil-dwelling amoeba *Dictyostelium discoideum*, a lower eukaryotic model organism.

Jaipargas et al. provide new data on the interaction of plant peroxisomes with mitochondria. Using live cell imaging, they show that peroxisomes form thin membrane protrusions (peroxules) which interact with mitochondria as a result of high light irradiation and subsequent ROS production. These interactions with ROS-distressed mitochondria may provide factors to peroxisomes which facilitate their proliferation for enhancing the ROS-combating capability of a plant cell. In line with this, Lismont et al. review our current view on redox-interplay between peroxisomes and mitochondria in mammalian cells. The authors outline the pro- and anti-oxidant systems of both organelles, their role as redox signaling nodes and discuss emerging evidence that peroxisomes and mitochondria share an intricate redox-sensitive relationship and cooperate in cell fate decisions. Schönenberger and Kovacs critically explore how hypoxia-inducible factor (HIF-α) signaling regulates the abundance and function of major oxygen-consuming organelles such as mitochondria and peroxisomes.

The reviews and research articles presented in this special topic demonstrate the impressive breadth of research currently being undertaken to understand the molecular mechanisms and physiological significance of organelle interactions and cooperation. Advances in the field, both methodological and conceptual, will have profound implications for understanding the architecture, organization and regulation of cellular metabolic and signaling networks and their impact on health and disease. Future challenges in this research area are to identify and characterize the specific components of the individual organellar interaction sites and to unravel signaling pathways which are able to dynamically regulate these interorganellar contact systems.

## Author contributions

MS and MI discussed and planned the content of the Editorial and wrote the manuscript.

### Conflict of interest statement

The authors declare that the research was conducted in the absence of any commercial or financial relationships that could be construed as a potential conflict of interest.

